# Feasibility and acceptability of HIV self-testing among pre-exposure prophylaxis users in Kenya

**DOI:** 10.7448/IAS.20.1.21234

**Published:** 2017-02-08

**Authors:** Kenneth Ngure, Renee Heffron, Nelly Mugo, Kerry A. Thomson, Elizabeth Irungu, Njambi Njuguna, Lawrence Mwaniki, Connie Celum, Jared M. Baeten

**Affiliations:** ^a^ Department of Public Health, Jomo Kenyatta University of Agriculture and Technology, Nairobi, Kenya; ^b^ Department of Global Health, University of Washington, Seattle, WA, USA; ^c^ Department of Epidemiology, University of Washington, Seattle, WA, USA; ^d^ Departments of Medicine, University of Washington, Seattle, WA, USA; ^e^ Center for Clinical Research, Kenya Medical Research Institute, Nairobi, Kenya

**Keywords:** Feasibility, acceptability, HIV, self-testing, PrEP, Kenya

## Abstract

**Introduction**: HIV testing is key to the delivery of pre-exposure prophylaxis (PrEP): testing HIV-uninfected at-risk persons is the first step for PrEP initiation and ongoing HIV testing is an essential part of PrEP delivery. Thus, novel and cost-effective HIV-testing approaches to streamline delivery of PrEP are urgently needed. Within a demonstration project of PrEP for HIV prevention among high-risk HIV serodiscordant couples in Kenya (the Partners Demonstration Project), we conducted a pilot evaluation of HIV self-testing.

**Methods**: Clinic visits were scheduled quarterly and included in-clinic HIV testing using fingerstick rapid HIV tests and refills of PrEP prescriptions. HIV oral fluid self-test kits were provided for participants to use in the two-month interval between scheduled quarterly clinic visits. Acceptability of HIV self-testing was assessed using both quantitative and qualitative methods.

**Results**: We found that 222 of 226 (98%) HIV-uninfected persons who were offered accepted self-testing. Nearly all (96.8%) reported that using the self-testing kit was easy. More than half (54.5%) reportedly did not share the HIV results from self-testing with anyone and almost all (98.7%) the participants did not share the HIV self-testing kits with anyone. Many participants reported that HIV self-testing was empowering and reduced anxiety associated with waiting between clinic HIV tests.

**Conclusions**: HIV self-testing was highly acceptable and may therefore be a feasible strategy to efficiently permit routine HIV testing between PrEP refills.

## Introduction

Pre-exposure prophylaxis (PrEP), in which a HIV-uninfected individual uses an antiretroviral (ART) medication to protect against HIV acquisition, has demonstrated efficacy for HIV protection in multiple geographic and at-risk populations worldwide [1–3], and World Health Organization (WHO) guidance recommends PrEP for persons at substantial risk of HIV infection [4]. PrEP use requires regular HIV testing - at the time of PrEP initiation and then on an ongoing basis - to reduce the risk of ART resistance if an HIV-negative person becomes HIV positive while using PrEP. This could occur, for instance, among people who use PrEP inconsistently and do not benefit from its protective effect. Clinical trials of PrEP, which rigorously evaluated its safety and efficacy, had monthly clinic visits that included HIV serologic testing. However, delivery models of PrEP are unlikely to uphold a monthly visit schedule because of excessive burden to PrEP takers and providers. For public health programmes, strategies to facilitate routine HIV testing are needed that reduce the burden to PrEP users and prescribers.

The acceptability of HIV self-testing has increased substantially in the last few years [5–16]. In 2012, the US FDA approved use of the oral fluid OraQuick In-Home HIV Test as the first self-administered test for HIV [17]. Several additional self-testing assays are now under development [18]. Studies from a variety of settings and the recently released HIV self-testing guidelines by WHO have demonstrated the potential of HIV self-testing to increase first-time and repeat testing for HIV [19]. In Africa, there is relatively limited experience with HIV self-testing, but some recent studies have showed high uptake and accuracy when self-testing was distributed to adults in the general population in Malawi and Kenya [5,15,16]. An additional study conducted in Kenya reported that HIV self-testing could increase male partner testing and promote safer sexual decision-making [20].

For persons using PrEP, regular HIV testing will be a standard part of delivering this prevention technology [21]. However, such testing will be relatively infrequent - quarterly in current guidance and potentially less frequently as PrEP delivery is further integrated into public health practice. Evaluating HIV self-testing as an adjunct to facility-based periodic testing among persons receiving PrEP may offer opportunities to improve the cost- and time-efficiency of laboratory monitoring of PrEP. Within a demonstration project delivering PrEP for HIV prevention, we conducted a pilot study of HIV self-testing. Our goal was to address key questions on feasibility, acceptability and use of HIV self-testing among HIV-uninfected persons initiating PrEP.

## Methods

### Setting, design and procedures

The Partners Demonstration Project is an open-label study of the integrated delivery of ART-based HIV prevention in which PrEP is offered as a bridge to ART therapy and viral suppression among 1013 high-risk HIV serodiscordant couples in 4 clinical research sites in Kenya and Uganda. PrEP is offered to the HIV-uninfected partner prior to and for the first six months after ART initiation by the infected partner, when viral suppression is expected. HIV serodiscordant couples are followed for up to twenty-four months [22]. We used a mixed-methods design [23] to prospectively study HIV self-testing behaviours among HIV-uninfected persons at the Thika, Kenya site, where 332 couples were enrolled. The eligibility criteria for this HIV self-testing sub-study included being HIV uninfected, and current use of PrEP and subset of those that enrolled in the HIV self-testing sub-study were selected through stratified purposeful sampling to participate in qualitative interviews. We aimed to interview 30 participants and therefore generated a list of 20 male and of 10 female participants (based on skewed gender of the HIV-uninfected participants enrolled in the Partners Demonstration Project and subsequently in HIV self-testing sub-study) and managed to reach saturation after interviewing 16 male and 7 female participants.

The HIV self-testing sub-study commenced in November 2013 and data for this analysis were complete as of June 2015. The Partners Demonstration Project was initiated in November 2012 and thus for most participants, enrolment into this sub-study occurred at a follow-up visit rather than at enrolment into the main study. Procedures for the sub-study were in addition to the routine procedures conducted for the Partners Demonstration Project.

The Partners Demonstration Project scheduled in-clinic HIV serologic testing (using the Kenya national rapid test algorithm of blood obtained by fingerstick or phlebotomy) at months 1, 3, 6, 9, 12, 15, 18, 21 and 24 after enrolment. For this HIV self-testing sub-study, self-testing was suggested to occur once per month, coinciding with opening a new bottle of PrEP medication during months when in-clinic testing was not scheduled, that is at months 2, 4, 5, 7, 8, 10, 11, 13, 14, 16, 17, 19, 20, 22 and 23. Participants were provided with a sufficient number of self-testing kits to conduct monthly testing until their next scheduled visit (i.e. two test kits in-between quarterly visits), and participants were counselled to use the self-testing kit at a place and time where they felt comfortable performing the testing (e.g. at home). At the time of enrolment into the HIV sub-study, participants performed one OraQuick® test on themselves, including interpretation of the test result under the guidance of the study staff. We used OraQuick information sheet to develop a more simplified pictorial information brochure which was then translated into local languages was provided. Participants were also provided with information about a toll-free 24-h helpline to call in case of challenges in performing the self-testing or in the event of a positive test result. This helpline was staffed by study counsellors. Participants were informed that any positive self-test result had to be confirmed by study staff in accordance with the Kenyan testing algorithm.

#### Quantitative data collection and analysis

Quantitative data collection occurred during the in-clinic quarterly visits via standardized interviewer-administered questionnaires about self-report of the number of tests performed, ease of performing the test, sharing of self-test kits and results, use of the 24-h helpline and preferences for oral versus finger prick for testing and self- versus clinic-based testing. The same questions on acceptability and use were asked at each quarterly visit. Descriptive analyses, conducted using SAS 9.4 (Cary, NC), were used to summarize data on the acceptability and use of HIV self-testing for all visits.

#### Qualitative data collection and analysis

In-depth interviews and focus group discussions (FGDs) were conducted using semi-structured guides by trained social scientists acting in the roles of facilitator and note taker. The FGDs were conducted separately for men and women. The goal was to gain a deeper understanding of participants’ experiences with HIV self-testing, including challenges. Qualitative discussions were recorded, transcribed and translated into English by the study team. The transcripts were reviewed separately by at least two investigators for completeness and initial theme generation. Coding and analysis was performed with Atlas.ti 6.2 software [24], using inductive and deductive approaches [25]. We then reviewed the results of our coding for consistency of text segmentation and code application with continued inter-coder agreement, and inconsistent results were reviewed by the coders until a consensus was reached and then the codes were grouped together into themes through consensus among the coders.

#### Assessing social harms

We assessed social harms in a triangulated fashion, through a review of counsellors’ chart notes, qualitative interviews and a review of the toll-free telephone notes. Social harms were also assessed quantitatively on a quarterly basis in the Partners Demonstration Project where all HIV self-testing participants were also enrolled.

### Human subjects

Ethical approval for the sub-study was obtained from the Kenyatta National Hospital Ethical Review Committee and University of Washington Human Subjects Division. All participants provided written informed consent.

## Results

### Participant characteristics

**Figure 1.**
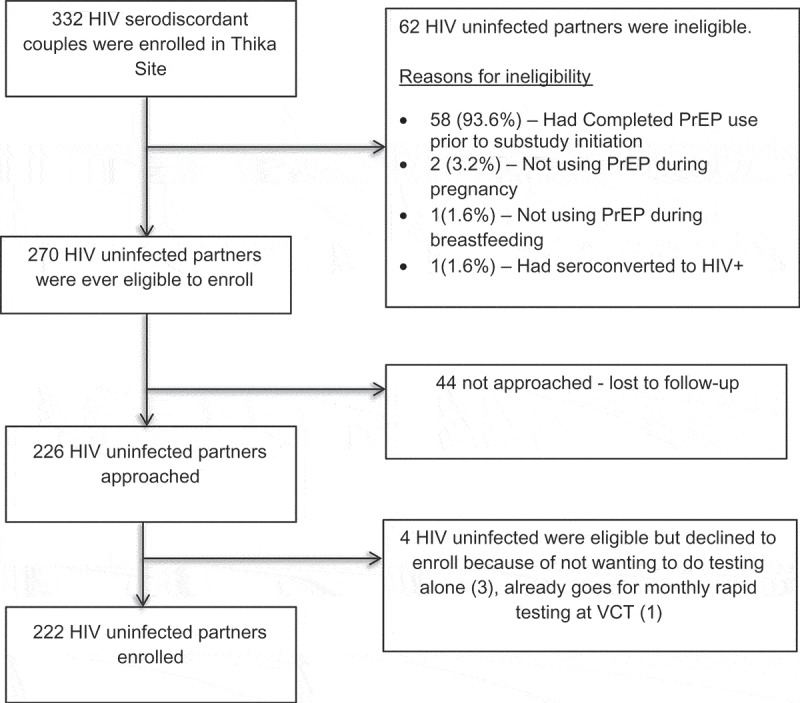
Flowchart of eligibility and enrolment status among Partners Demonstration Project participants at Thika site.

Among the 332 HIV-uninfected members of HIV serodiscordant couples enrolled at Thika site, 264 were eligible for the HIV self-testing sub-study, of whom 226 were approached and 222 (98.2%) enrolled (Figure 1).

Three-quarters of the participants enrolled were men (*n* = 177) and 45 were women, reflecting the general distribution of subjects receiving PrEP in the Partners Demonstration Project at the Thika site. The median age was thirty years and number of years of education was 8.5 (Table 1).

**Table 1.** Participant characteristics, frequency (%) or median (IQR)

	Male	Female	Total
Participants enrolled	177	45	222
Month of enrolment, median (IQR)	3 (3–6)	3 (1–6)	3 (3–6)
Age, median (IQR)*	30 (27–37)	32 (27–38)	30 (27–37)
Married to study partner*	168 (94.9)	45 (100.0)	213 (95.6)
Number of children, median (IQR)*	1 (0–2)	2 (1–3)	1 (0–2)
School completed (years), median (IQR)*	10 (8–12)	8 (7–11)	8.5 (8–12)
Has any monthly income*	170 (96.1)	34 (75.6)	204 (91.9)
Number of sex acts with study partner during month prior to enrolment, median (IQR)*	8 (4–12)	7.5 (2–12)	8 (3–12)
Had unprotected sex with study partner during month prior to enrolment*	45 (25.4)	10 (22.2)	55 (24.8)
Had additional sexual partner(s) during month prior to enrolment*	8 (4.5)	0	8 (3.6)

*Baseline data obtained at enrolment to the Partners Demonstration Project.

Participants were enrolled into the HIV self-testing sub-study, a median of three months (Interquartile range [IQR] 3–6) after enrolment into the Partners Demonstration Project. Qualitative data collection included 23 in-depth interviews (16 men, 7 women) and 7 FGDs (4 among men and 3 among women with a total of 32 men and 24 women).

### Acceptability of HIV self-testing

Of those enrolled in this sub-study, 219 had at least 1 follow-up visit and 93.2% of those reported conducting HIV self-testing at least once. Across all visits, a total of 1225 HIV self-testing kits (95.6% of 1282 dispensed) were reported to have been used, 253 (98.1%) and 972 (94.9%) among HIV-uninfected women and men, respectively. The median follow-up time in the HIV self-testing sub-study was eleven months (IQR 8.3–13.9).

### Use of HIV self-testing

A majority (96.8%) of participants reported that using the HIV self-testing kit was “easy” or “very easy”. Most participants (90.8%) reported that they did not receive any help with performing the test (Table 2).

**Table 2.** HIV self-tests conducted at all visits by HIV-uninfected participants on PrEP.

	All visits, frequency (%)		
Testing uptake	Male (*n* = 515)	Female (*n* = 136)	Total (*n* = 651)
**Participant’s self-testing coincide with anything**			
*Did not coincide with anything*	278 (54.0)	69 (50.7)	347 (53.3)
*Opening a new bottle [of PrEP]*	226 (43.9)	65 (47.8)	291 (44.7)
*Unprotected sex*	12 (2.3)	0	12 (1.8)
*Other*	0	2 (1.5)	2 (0.3)
**Participants ease in performing the test**			
*Difficult*	1 (0.2)	1 (0.7)	2 (0.3)
*Not difficult but not easy*	16 (3.1)	3 (2.2)	19 (2.9)
*Easy*	329 (63.9)	101 (74.3)	430 (66.1)
*Very easy*	169 (32.8)	31 (22.8)	200 (30.7)
**Presence of another person when participant used the self-test kit**			
*No one*	332 (64.5)	109 (80.2)	441 (67.7)
*Study partner*	178 (34.6)	26 (19.1)	204 (31.3)
*Children*	0	2 (1.5)	2 (0.3)
*Friend*	1 (0.2)	0	1 (0.2)
*Other*	1 (0.2)	0	1 (0.2)
**Received help with HIV self-testing**			
*No one*	469 (91.1)	122 (89.7)	591 (90.8)
*Study partner*	46 (8.9)	12 (8.8)	58 (8.9)
*Children*	–	2 (1.5)	2 (0.3)
**Shared HIV test results**			
*No one*	280 (54.4)	75 (55.2)	355 (54.5)
*Study partner*	231 (44.9)	58 (42.7)	289 (44.4)
*Others (parents, children, friends, siblings)*	4 (0.8)	1 (0.7)	5 (0.8)
**Shared HIV self-test kits with others**			
*No*	507 (98.5)	136 (100.0)	643 (98.7)
*Yes*	8 (1.5)	0	8 (1.3)
**Participants preferred location for HIV testing**			
*Self-testing only*	253 (49.3)	86 (63.2)	339 (52.2)
*Clinic-based testing only*	48 (9.4)	9 (6.6)	57 (8.8)
*Sometimes self-testing and sometimes clinic testing*	212 (41.3)	41 (30.2)	253 (39.0)
**Participants preferred method for HIV testing**			
*Rapid test with blood from finger prick*	50 (9.8)	10 (7.4)	60 (9.2)
*Oral fluid testing*	276 (53.8)	92 (67.7)	368 (56.7)
*No preference*	187 (36.5)	34 (25.0)	221 (34.1)

A minority (8.9%) received help from their study partners. A total of 17 calls were made to the 24-h helpline in relation to challenges in performing or interpreting the test results. Only one participant called to report a preliminary positive test, which was not confirmed on follow-up testing at the nearest public HIV testing centre on the same day or at the research site during the subsequent study visit.

More than half of the participants (53.3%) reported that HIV testing did not coincide with any specific activity while 44.7% of participants reported that they self-tested when they opened a new bottle of PrEP and 1.8% reported testing after unprotected sex. The majority (67.7%) of participants reported testing alone, while close to a third (31.3%) reported that their study partner was with them. More than a half (54.5%) of the participants reported that they did not share their test results with anyone, while 44.4% reported sharing the HIV self-testing results with their study partner. Additionally, almost all (98.7%) the participants reported not sharing the HIV self-testing kits with anyone.

### Participant experiences with HIV self-testing

In the context of PrEP use, more than half (52.2%) of the participants reported that they would prefer HIV self-testing only, 8.8% would prefer clinic-based testing by a health provider while a 39.0% would prefer a mix of self-testing and provider testing, the model used in the Partners Demonstration Project. More than half (56.7%) of the participants preferred the oral testing while 9.2% preferred the finger prick and a third (34.1%) did not indicate any preference of HIV testing method (Table 2).

In the qualitative interviews, a key motivation for accepting HIV self-testing was that it reduced the anxiety associated with waiting to return to the clinic for scheduled facility-based testing.

Every day, every time thinking to yourself, how will it be when I go back there? When you test yourself, you know your status; you relax. (Female respondent 2, FGD 01)

In line with the quantitative surveys, most participants reported that HIV self-testing was easy, even when they performed it for the first time. Only one respondent reported that it was initially difficult to conduct the HIV self-testing at home and cited that the first time she accidentally spilled the buffer and had to use the buffer allocated for the following month. While most did not use the helpline because they were able to recall and execute the self-testing procedure without any problems, they acknowledged that the helpline was a useful support that needed to be put in place in case of challenges in conducting or interpreting the HIV self-test results.

We first tested with him/her (provider). He/She first tested me, and then he/she gave me mine to go test at home. If two lines appear I stop taking medicine, I call them (clinic nurses). And I felt it was simple. (Female respondent 7, FGD01)

It doesn’t have a lot of work. You know like blood draw; you know sometimes it can be burdensome … You know sometimes for blood draw it can happen you [referring to himself] prick yourself wrongly. And you have nothing to prevent germs from getting in … But this one (oral kit) is easy. (Male respondent 3, FGD 02)

When asked, what reminded them to self-test, respondents reported using personal reminders such phone alarm, keeping the self-testing kit next to their bottle(s) of PrEP and receiving verbal reminders from their study partners. Travel was one explanation for not testing when opening a new bottle of PrEP, since the self-testing kit was relatively large and cumbersome to carry out of town.

*We were just given two. When the first medicine* [bottle of PrEP] *is over, you test. When the second medicine is over you test. (Female respondent, IDI 03)*

Someone tests … on the date you are told. The date has been written for you. No, I cannot forget. If I cannot forget to take medicine, even that one I cannot forget. (Female respondent, FGD 01)

Many respondents reported testing alone and if the partner was not present when they performed the HIV self-testing, they would later share the results with them. Some reported saving the testing device with the results displayed for their partners to see. Among those who did not share their results, some respondents reported that they would not have minded if their partner learned of their results since they were already aware of their serodiscordant status. Additionally, most of the participants reported that they had received several HIV-negative tests and hence an additional negative test result was not surprising especially since they were also adherent on PrEP. One female respondent reported that she asked her partner to keep track of the ~15 min before reading the result while she attended her household chores.

I test when she is there. Since I came here I tell her my results. Eeeh. I call her so that she does not give me a divorce. (Male respondent 01, FGD02)

No, I test alone. Mmmh. You know sometimes she is at work, so the time I get to go home … I calculate. It is just 20 minutes and I finish, you know sometimes she is far…. (Male respondent, FGD02)

It is not good for children to know our status because they can go out to play and then shout saying, “You know daddy and mommy are like this and that….” (Male respondent, FGD 02)

Regarding sharing of HIV self-testing kits, some men reported that their female HIV-infected partners had wanted to use their oral HIV self-testing kits independently confirm they were truly HIV infected. Another respondent reported that at one time, her friend found her testing herself and she explained what the kit was for. When the friend got interested and wanted to test, the respondent declined because she felt she was not able to counsel. Another respondent described giving one of her kits to the family doctor who was curious to find out how it works. Respondents suggested that if they could be given at least one additional kit per visit, it would allow them to share with their partners and other people close to them, including their children.

I have once found that she has used one kit (Respondents and moderator laugh). She tested herself. She came and told me that she has used one, I did not reprimand her because she wanted to know her status. But I told her not to use [HIVST kit] again. (Male respondent, FGD 05)

But to tell you the truth, if I am given more test kits, I would like to test my wife to confirm her HIV status. I still do not believe that my wife is HIV positive. I am negative, the children are negative and my wife is positive. (Male respondent, IDI 04)

Many respondents reported that they preferred self-testing compared to provider testing because of confidentiality, ease of use and convenience. For those who preferred health facility testing, they cited the advantage of having a provider nearby to give support in the event that their test result was positive. Many expressed preferences for oral testing, rather than a finger prick (as is done in the clinic) because the latter was more painful while others reported no specific preference because they were comfortable with both approaches. Some reported that they initially did not trust the self-testing kit, but after using it several times, they now trusted the results. One female respondent had initially declined to perform oral HIV self-testing at home because she was afraid to do it on her own in the event that the test was positive.

With HIV, self-testing you do not require any bus fare, anytime you can just test yourself. (Male respondent, IDI 04)

I am saying … I find all of them to be good. When you get used, after the doctor has explained to you…. The way I was explained to … I understood and felt that all (modes of testing) are okay. (Male respondent, FGD 02)

May be if those doubts someone has them, it is not working as it should…. You know … to many people … they know blood is the accurate one. But you know this one (self-test kit) doesn’t have any blood. (Male respondent, FGD 02)

There were no intended social harms (including physical, psychological, social and economic) reported as a consequence of conducting the HIV self-testing or sharing the test results.

## Discussion

Within a prospective study of Kenyan HIV serodiscordant couples receiving PrEP, we evaluated HIV self-testing as a model for HIV testing between scheduled clinic visits. We found high (98.2%) acceptability and continuation of HIV self-testing, and participants reported that self-testing offered convenience, was easy to use and could be used confidentially. These pilot results suggest that self-testing may be used to facilitate HIV testing and potentially reduce the frequency of clinic visits for HIV testing during PrEP delivery.

A majority of both male and female participants in both the quantitative and qualitative interviews reported that conducting and interpreting the HIV self-testing results was easy and most reported that they did not get any help conducting the test. Similar findings have been reported in multiple published studies of HIV self-testing used for HIV diagnosis from diverse settings [1,5], including Kenya [15,16,20,26]. Clear instructions were provided in this project and in the participants’ preferred language, which may have facilitated execution of the test. Few participants accessed the helpline to report challenges in conducting the test or to seek assistance with positive results. Given the very low HIV incidence observed in this cohort, and in general among persons taking PrEP in open-label studies, this is not surprising and may indicate a context in which HIV self-testing could be readily implemented. However, the low volume of calls to the helpline experienced in our study may be lower than in programmatic delivery, unless such programmes are able to put in strong support measures including, training coupled with demonstrations on HIV self-testing.

HIV self-testing enables people to test themselves discreetly and conveniently which is important to people who have concerns about confidentiality and who are not currently reached by existing HIV testing and counselling services [7,8]. In our study, more than a half of the participants reportedly did not share their test results with anyone including their study partners, possibly because they had undergone couples HIV counselling and testing and therefore knew each other’s status. For PrEP, home-based HIV self-testing has the potential to assuage concerns about incident HIV between scheduled in-clinic testing appointments or even to reduce the frequency of in-clinic HIV testing. The frequency of clinic-based HIV testing is a major contributor to PrEP delivery costs, both direct costs to health systems (because of clinic staffing and burden to busy facilities) and opportunity costs to patients (travel, time off work etc.). HIV testing in PrEP delivery has evolved from the monthly testing provided in clinical trials, to successful implementation of quarterly testing in demonstration projects and clinical practice. Although not tested directly in this study, it is possible that self-testing might allow even longer time between clinic visits while still being feasible, acceptable and safe, particularly as HIV incidence appears to be very low in persons taking PrEP with sufficient adherence.

The sensitivity of oral fluid self-testing to detect HIV infection is slightly lower than finger stick blood-based testing [27]. In the setting of initial HIV testing, such as when initiating PrEP, oral fluid tests are not recommended, given lower sensitivity than blood-based tests and potential for false-negative results. However, incident HIV infection is rare among persons taking PrEP with sufficient adherence, and self-testing, even with lower sensitivity, would have greater detection of infection than no testing in between clinic visits.

To our knowledge, this is the first study to evaluate acceptability and use of HIV self-testing among persons using PrEP. In addition, our study utilized both quantitative and qualitative methods to gather data on acceptability and performance of self-testing - we used qualitative findings to explore the quantitative findings [23]. The prospective design of our study was able to demonstrate acceptability and use of HIV self-testing over time.

Our study was conducted exclusively among HIV-uninfected partners in mutually-disclosed HIV serodiscordant relationships who had known they were recently HIV uninfected based on clinic-based rapid testing plus were taking open label PrEP and thus may have had very low expectation of getting a positive result - which may limit generalizability of our findings. Self-testing was conducted essentially monthly by participants in this sub-study. While monthly testing is more than is recommended for PrEP delivery, the approach used tested a model of alternating in-clinic testing and HIV self-testing conducted by participants at home. Future research could explore a model of quarterly HIV self-testing and six monthly clinic visits for PrEP refills with clinic based HIV testing for stable PrEP users, with an allowance for interim visits for those with positive/invalid tests or those with acute HIV seroconversion symptoms.

## Conclusions

In sum, HIV self-testing was found to be highly acceptable to Kenyan men and women using PrEP and could therefore be a suitable adjunct to PrEP delivery in this population. Incorporating HIV self-testing in PrEP delivery settings could provide an efficient method that has the potential to reduce participant and health provider burden associated with frequent facility-based HIV testing for established PrEP users. Next steps, therefore, could include using HIV self-testing to streamline PrEP delivery - specifically through decreasing the frequency of PrEP follow-up clinic visits, by alternating HIV testing in the clinic with HIV self-testing at home.
